# Chemical castration in dogs using calcium chloride: effects on testicular hemodynamics and semen characteristic and serum levels of testosterone

**DOI:** 10.1186/s12917-024-04353-9

**Published:** 2024-11-11

**Authors:** Alaa Mohamed, Mohamed Fathi, K. H. El-Shahat, Ashraf A. Shamaa, Mohamed M. Bahr, Mohamed A. El-Saied

**Affiliations:** 1 Theriogenology Department, Faculty of Veterinary Medicine, Cairo University, Giza, 12211 Egypt; 2 Surgery, Anesthesiology and Radiology Department, Faculty of Veterinary Medicine, Cairo University, Giza, 12211 Egypt; 3 Department of Pathology, Faculty of Veterinary Medicine, Cairo University, Giza, 12211 Egypt

**Keywords:** Canine, Doppler, Calcium chloride, Sperm, Testicular artery, Volume

## Abstract

Dog overpopulation and stray dogs are global issues that are detrimental to public health and animal welfare. Thus, the goal of the current study was to provide alternatives for surgical castration. Therefore, calcium chloride was employed in this study, which might be an option for castration. Ten dogs were divided into two groups of five: a calcium chloride-treated group and a control group. The treated group received a single bilateral intratesticular injection of 1 ml of sterile saline containing calcium chloride dihydrate (CaCl2•2 H2O) at a dose of 20 mg/kg per testicle. While the control group was treated with 1 ml of sterile saline solution, Semen and blood collection, as well as Doppler ultrasonography, were routinely carried out every week on days 0, 7, 14, 21, and 28 in order to evaluate the impact of the injection on semen parameters and testicular blood flow. The testicular volume and echogenicity in the CaCl2-treated group were significantly (*P* < 0.001) lower in weeks 2 through 4 than in the control group. Furthermore, in canine semen, CaCl2 dramatically decreased the amount, motility, and viability of sperm. When compared to vehicle-control animals, azoospermia was seen 2 weeks after the injection and persisted for the end of the study. The testes of all dogs were surgically removed at 30 days post-injection, and testes were put in 10% neutral buffered formalin for tissue processing. When compared to the control group, the average weight of testes in the chemical groups was dramatically reduced. Significant decreases in spermatogenic processes, necrosis, and degeneration of seminiferous tubules packed with necrotic debris, and fibrosed interstitial tissue, necrosed and calcified Sertoli, and Leydig cells were seen 30 days after CaCl2 injection. There was a significant decrease in testosterone levels compared to day 0 before CaCl2 injection and the control group. From weeks 1 through 4, there was a substantial decrease in both peak systolic velocity (PSV) and end-diastolic velocity (EDV) values (*P* < 0.001) following a single intratesticular injection of CaCl2. The resistance index (RI) and pulsatility index (PI) showed the opposite tendency. Based on the histopathological and semen evaluations in this investigation, the study concludes that a single intratesticular injection of CaCl2 appears to be a practical and generally applicable approach for chemical sterilization of dogs.

## Introduction

A large number of stray dogs in Egypt have become a serious public health concern since aggressive dog colonies are multiplying quickly and dispersing illnesses such as rabies. There have been instances of stray dogs attacking animals in rural regions [1]. According to a government census, there are almost 4 million stray dogs in Cairo. The Egyptian Federation for Animal Welfare (EFAW) organized seminars in 2011 in response to this rising issue. EFAW worked with veterinary directorates to promote humane solutions, such as surgical sterilization (castration), to regulate the stray dog population. Despite being successful, surgical sterilization is expensive, necessitates the expertise of medical professionals, and carries risks of bleeding, infection, and the need for complicated and time-consuming post-operative care [2].

Over the past forty years, institutions, organizations, and businesses throughout the world have been compelled by the overpopulation of dogs and cats to investigate non-surgical sterilization options. Injections of steroid hormones and gonadotropin-releasing hormone (GnRH) agonists and antagonists are two temporary techniques for controlling fertility. Immunizations against GnRH, egg/sperm proteins, and gonadotropins have also been investigated. Intratesticular injections of chemical agents such as glycerol, lactic acid, calcium chloride, arginine, zinc, and carboxylic acid derivatives are used in permanent procedures. The ACC&D report (2013) has extensive details. Chemical substances that cause seminiferous tubule destruction by burning or osmotic processes are the method via which chemical castration works. Sodium chloride (NaCl), lactic acid, and calcium chloride (CaCl2) are notable chemical castrators [3–5]. This testicular injection technique provides a less invasive castration choice. However, research indicates that for actual chemical castration, CaCl2 works better than sodium chloride [6]. A notable study showed that a powerful chemical method for sterilizing male canines is a single intratesticular injection of CaCl2 [7, 8].

Testicular dimensions and echogenicity were significantly reduced as a result of the injection, and there was also significant testicular necrosis. This is crucial because the testes are highly metabolic organs that depend on a steady flow of blood to maintain spermatogenesis and steroidogenesis [9]. For testicular function to be at its best, blood perfusion must be well controlled [10]. Testicular blood flow can be affected by variables including age, diet, health, environment, and hormone balance [11]. However, the impact of a CaCl2 intratesticular injection on testicular hemodynamics has not been investigated in any research studies. Thus, the goal of this research is to find out how a single intratesticular injection of CaCl2 affects the hemodynamics of the testicles and the characteristics of the semen in male stray dogs.

## Materials and methods

### Experimental location and ethical approval

This study was conducted at the dog stud of the Department of Theriogenology, Faculty of Veterinary Medicine, Cairo University (latitude 30°01’ N; longitude 31°21’ E), from October 1, 2023, to November 1, 2023. The study received ethical approval from the institutional animal care committee of the Faculty of Veterinary Medicine, Cairo University (Approval No.: Vet CU 18042024892).

### Animals and management

The study involved ten male stray dogs of unknown breeding history, aged 2 to 3 years, and weighing 25 kg on average. Prior to treatment, monthly semen collections were performed to ensure all dogs exhibited excellent fertility through semen analysis. The dogs were housed in a dedicated room at Cairo University’s Theriogenology Department, provided with individual boxes, fed a diet of commercial food comprising cereals, fats, vegetables, and vitamins, and had access to tap water. A comprehensive clinical examination was conducted, including genital palpation, testicular ultrasonography, semen collection, and blood sampling.

### Experimental design

Ten dogs were randomly divided into two groups of five: a CaCl2-treated group and a control group. The treated group received a single bilateral intratesticular injection of 1 ml of sterile saline containing CaCl2·2 H2O at a dose of 20 mg/kg per testicle [8, 12]. While the control group was treated with 1 ml of saline solution, routine examinations, including blood collection, semen collection, and doppler ultrasonography, were performed weekly on days 0, 7, 14, 21, and 28 to assess the effects of the injection on testicular blood flow and semen parameters, comparing the treated and control groups.

### Preparation and intratesticular injection of calcium chloride

The maximum dose of CaCl2 for induction of chemo-sterilization was 20 mg/kg body weight of animals in the present study according to Jana and Samanta [8]. The dose depends on the body weight of the dog (25 kg on average). So the calculated dose per testis is 500 mg. To prepare the solution containing CaCl2, 20 mg/kg body weight of animals, CaCl2 dihydrate powder (Sigma Aldrich Corporation) was added to 1 mL sterile normal saline 0.9 (FIPCO), mixed well. Then the solution was sterilized with a Millipore filter with 0.45 μm pores when used [13].

Following Silva et al. [14], xylazine (1.0 mg/kg) and ketamine (1.0 mg/kg) were injected intramuscularly to sedate the dogs. Before being injected, the testicles were sterilized with 70% ethyl alcohol. To ensure linear penetration of the fluid throughout the whole path as the needle was withdrawn, a sterile 21-gauge needle was introduced from the caudoventral aspect of each testis, approximately 1 cm from the epididymal tail [8].

### Semen collection and evaluation

Semen was collected using manual manipulation as reported by Linde-Forsberg [15], with ejaculates collected into a pre-warmed cup (36–38 °C). This process worked best when an estrous bitch was close. The dog’s penis was initially vigorously rubbed via the prepuce at the level of the bulbus glandis until a partial erection showed. After that, the penis was squeezed between the thumb and index finger, and a complete erection was achieved by pelvic thrusting. The semen samples were sent to the lab for further examination after the first two fractions were collected.

The sperm motility, morphology, L/D ratio, and concentration of the first two fractions were assessed after collection. Using a light microscope (100 x and 400x magnification), a drop of semen was placed on a warmed microscope slide, and the progressive movement of sperm was estimated to the nearest 5% (LABOMED, Labo America, Inc., U.S.A.). The numbers of live and dead spermatozoa and spermatozoal morphology were evaluated by placing a small drop of semen on a warmed microscope slide and staining it with eosin-nigrosin.100–200 spermatozoa in total were counted under oil at 1000 x [16]. The concentration of sperm cells has been measured using a light microscope (400 x magnification) (LABOMED, Labo America, Inc., U.S.A.) and a hemocytometer (Neubauer counting chamber) [17].

### B-mode ultrasonography examination

Both testes were ultrasonically examined in each dog using a linear array transducer (5–7.5 MHz). The transducer was positioned on the lateral surface of the testis, and acoustic gel was applied to the skin while the patient was in the dorsal recumbent posture. Using the mediastinum as a reference, testicular length and width were measured using longitudinal and transverse B-mode imaging. Normal, hypoechoic, and hyperechoic testicular parenchyma appearances were distinguished, as was the existence of abnormal echogenic stippling [18].

### Doppler ultrasonography

The ExaGo Doppler device (France) with a linear probe (5–7.5 MHz) was used to visualize the tortuous distal region of the supratesticular artery. The transducer was positioned near the scrotal neck, while noise and a pulse-wave Doppler gate were inserted inside the lumen of the vessel. In addition to measuring the peak systolic velocity (PSV) and end-diastolic velocity (EDV), the device software also computed the resistance index (RI) and pulsatility index (PI). When necessary, angle modifications were made, usually keeping the angle at 0° [18].

### Testicular histopathology

The dogs were surgically castrated at the end of the research while under xylazine and ketamine (1.0 mg/kg) intramuscular anesthesia. The right and left testes of all dogs in the control and treated groups were weighed after castration. Samples of testicles were persevered in 10% neutral buffered formalin. Specimens were collected 30 days post-inoculation with Cacl2. Formerly, they were processed using a tissue processor (HistoCore PEARL, Leica, Germany), then they were embedded in paraffin blocks and sectioned using microtome at 4 μm thickness. The tissue sections were stained with Hematoxylin and eosin (H&E) stain and examined via an Olympus BX43 light microscope connected to Olympus DP27 camera linked to CellSens Dimension software.

### Blood sampling

Two milliliters of blood were collected from the cephalic vein and centrifuged at 3,000 g for 5 min to extract serum [19]. Samples were taken an hour after administering Receptal^®^ (GnRH analogue) at 0.4 µg/kg body weight [20].

### Hormonal assessments

The serum testosterone level was measured using commercial enzyme immunoassay kits (ELISA) according to Taya [21].

### Statistical analyses

All data are presented as the mean ± standard error of the mean and were first checked for normality by the Kolmogorov-Smirnov test. Repeated measures ANOVA test was used for discrimination of the differences among the means in the studied time points, and finally, the Bonferroni post hoc test. GraphPad Prism5 software was used for all the statistical assessments studied. *P* < 0.05 was considered statistically significant.

## Results

All animals tolerated the intratesticular injections of CaCl2 well. In addition to a minor increase in testicular firmness upon palpation, they didn’t suffer from any significant inflammatory swelling or agitation fever. Within a minute or two of starting CaCl2, the majority of the dogs displayed signs of minor pain. All of the dogs showed signs of mild testicular swelling 24 h after injection. In treated animals, swelling increased 48 to 72 h after injection and subsequently progressively reduced after two weeks. Throughout the trial, every dog that received a CaCl2 injection lived and was in good health. Throughout the trial, the animals’ behavior did not significantly change.

### Changes in testicular weight

Following castration, a single intratesticular injection of CaCl2 significantly (*P* < 0.0001) reduced testicular weight compared to the control group. The weight of the testicles in the control group was 15.48 ± 0.98 gm (combined testes) and 5.380 ± 0.26 gm (combined testes) in the treated group.

### Histopathological changes in the testes

**Fig. 1:**
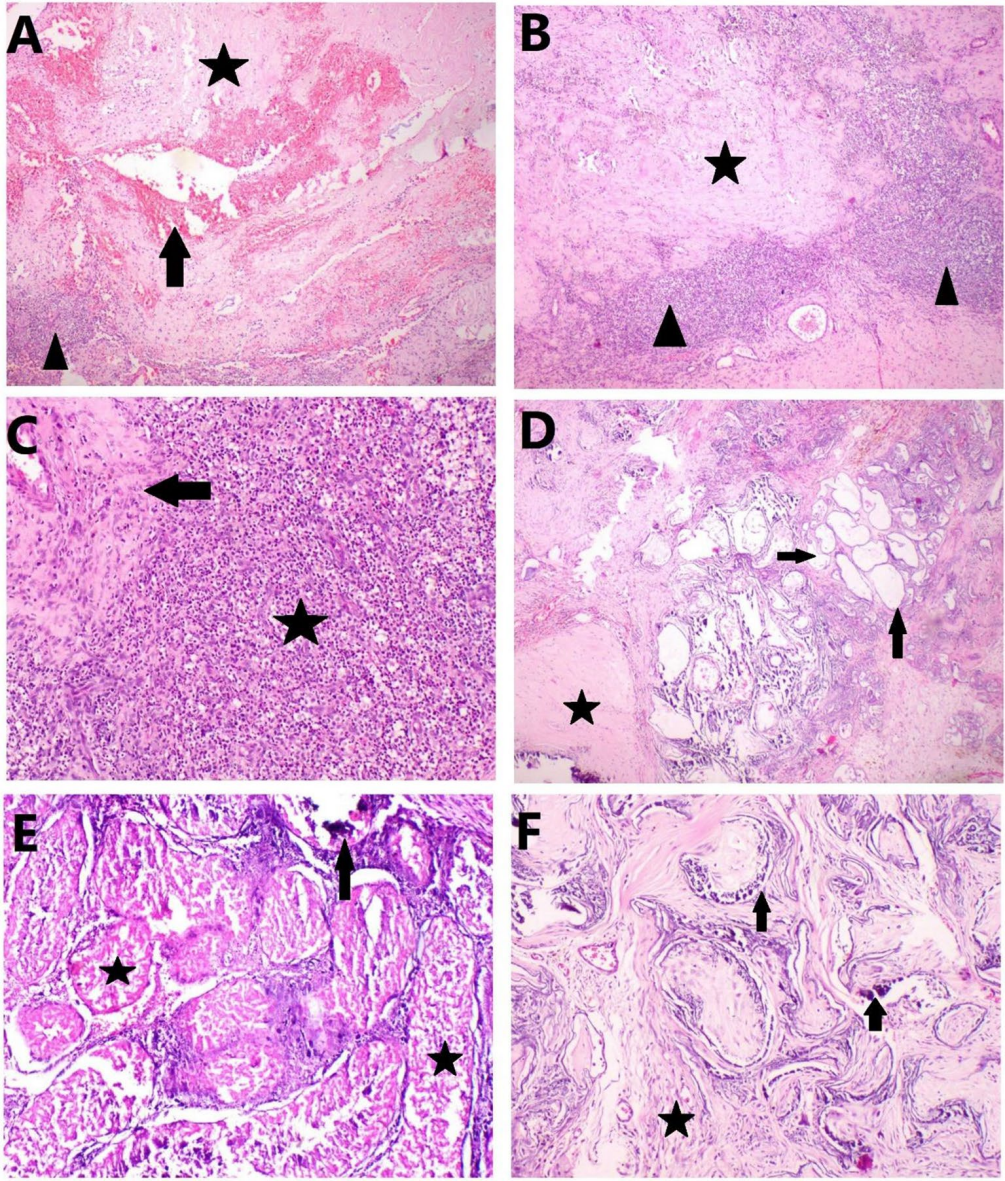
Photomicrograph of H&E-stained testicular tissue at 30 days post-inoculation: **A**) Extensive hemorrhage (arrow) and fibrosis (asterisk) of testicular section with mononuclear inflammatory cells infiltration (arrowhead), **B**) Complete loss of parenchymal architecture with abundant collagen deposition (asterisk) and mononuclear inflammatory cells infiltration (arrowhead), **C**) Higher magnification showing mononuclear inflammatory cells infiltration (asterisk) with interstitial collagen deposition (arrow), **D**) Cystic dilation of seminiferous tubules (arrow) with massive scirrhous reaction (asterisk), **E**) the lumen of seminiferous tubules filled with necrotic debris with complete loss of seminiferous tubules lining epithelium and Sertoli cells (asterisk) with beside accompanied by basophilic calcium salt deposition (arrow) and necrosis of Leydig cells, **F**) Fibrosis of interstitial tissue (asterisk) with calcification of necrosed seminiferous tubules (arrow) with fading of Leydig cells

The examined testicular sections showed marked defective and reduction of spermatogenic processes. The testicles were diffusely necrosed with extensive hemorrhage and fibrosis. The examined testicular sections showed abundant collagen deposition and complete loss of parenchymal architecture with a few remnants of seminiferous tubules suffering from cystic dilation. The lining epithelium of seminiferous tubules and Sertoli cells was completely necrosed accompanied by calcification. The interstitial tissue was fibrosed and infiltrated with mononuclear inflammatory cells beside Leydig cells were substantially necrosed (Fig. 1).

### B-mode ultrasonography findings

#### Volume of the testes

**Fig. 2:**
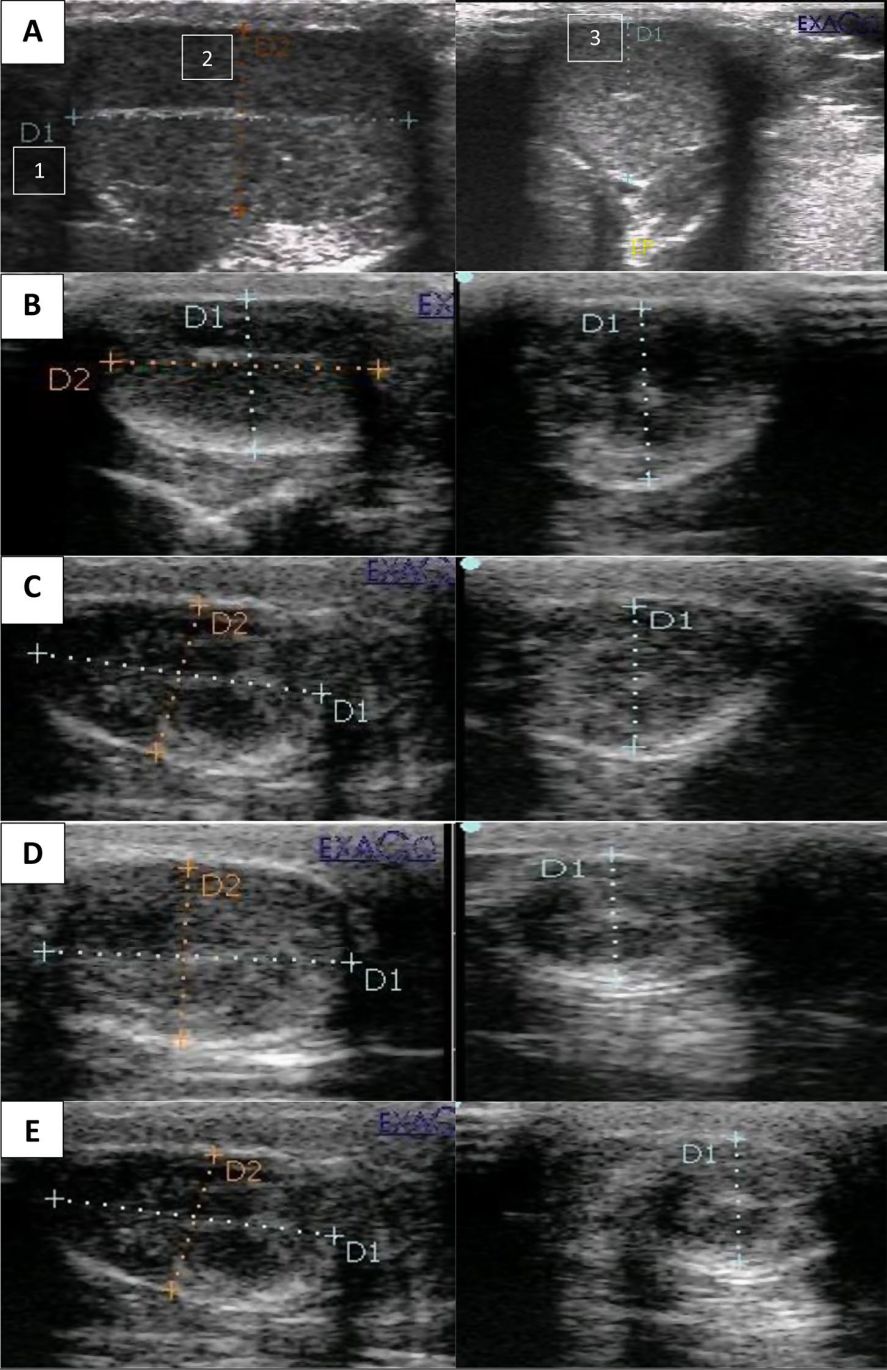
Ultrasonography revealed the testicular dimensions to estimate the testicular volume using the ellipsoid shape formula. Length (1) and height (2) are measured in the longitudinal scan, while the testicular width (3) is measured in the transverse scan before and after intratesticular injection of calcium chloride dihydrate. **A**: Measuring the length, height and width before injection. **B**, **C**, **D** and **E**: first, second, third and fourth weeks after injection, respectively. The diagram shows the comparison of the echogenicity and volume of the testes in the 0, first, second, third and fourth weeks post injection

There was a significant (*P* < 0.001) reduction in testicular volume in the CaCl2-treated group from weeks 2 to 4 compared to day 0 before CaCl2 injection and controls, with more pronounced reductions observed at weeks 3 and 4 (Fig. 2; Table 1).

**Table 1:** Effect of single intratesticular injection of calcium chloride on the volume of testes (cm^3^) (mean ± SEM) on day 0 before the injection of calcium chloride and 4 weeks post-injection

		Duration of study				
Groups	Traits	Day 0	1st weeks	2nd weeks	3rd weeks	4th weeks
	Volume of right testes	3.8 ± 0.1^ac^	3.9 ± 0.2 ^ac^	3.9 ± 0.2 ^ac^	4.0 ± 0.2 ^ac^	4.0 ± 0.2^ac^
Control						
	Volume of left testes	4.0 ± 0.1^ac^	4.0 ± 0.2 ^ac^	3.9 ± 0.1^ac^	4.0 ± 0.1^ac^	4.0 ± 0.1^a c^
	Volume of right testes	4.0 ± 0.2^ac^	3.4 ± 0.1^ad^	3.0 ± 0.1^bde^	2.7 ± 0.1^be^	2.6 ± 0.1^be^
Cacl2						
	Volume of left testes	4.0 ± 0.1^ac^	3.6 ± 0.1^ad^	3.1 ± 0.1^be^	3.2 ± 0.1^be^	3.0 ± 0.0 ^be^

Mean with different superscripts (a, b) within the same column, (c, d, e ) within rows were significantly different at *P* < 0.001

#### Echogenicity of the testes

Testes in the treated group showed a significant reduction in echogenicity over weeks 2, 3, and 4, with the lowest echogenicity noted at week 4 post-injection as compared to day 0 before CaCl2 injection and controls (Fig. 2).

### Doppler-mode findings

**Fig. 3:**
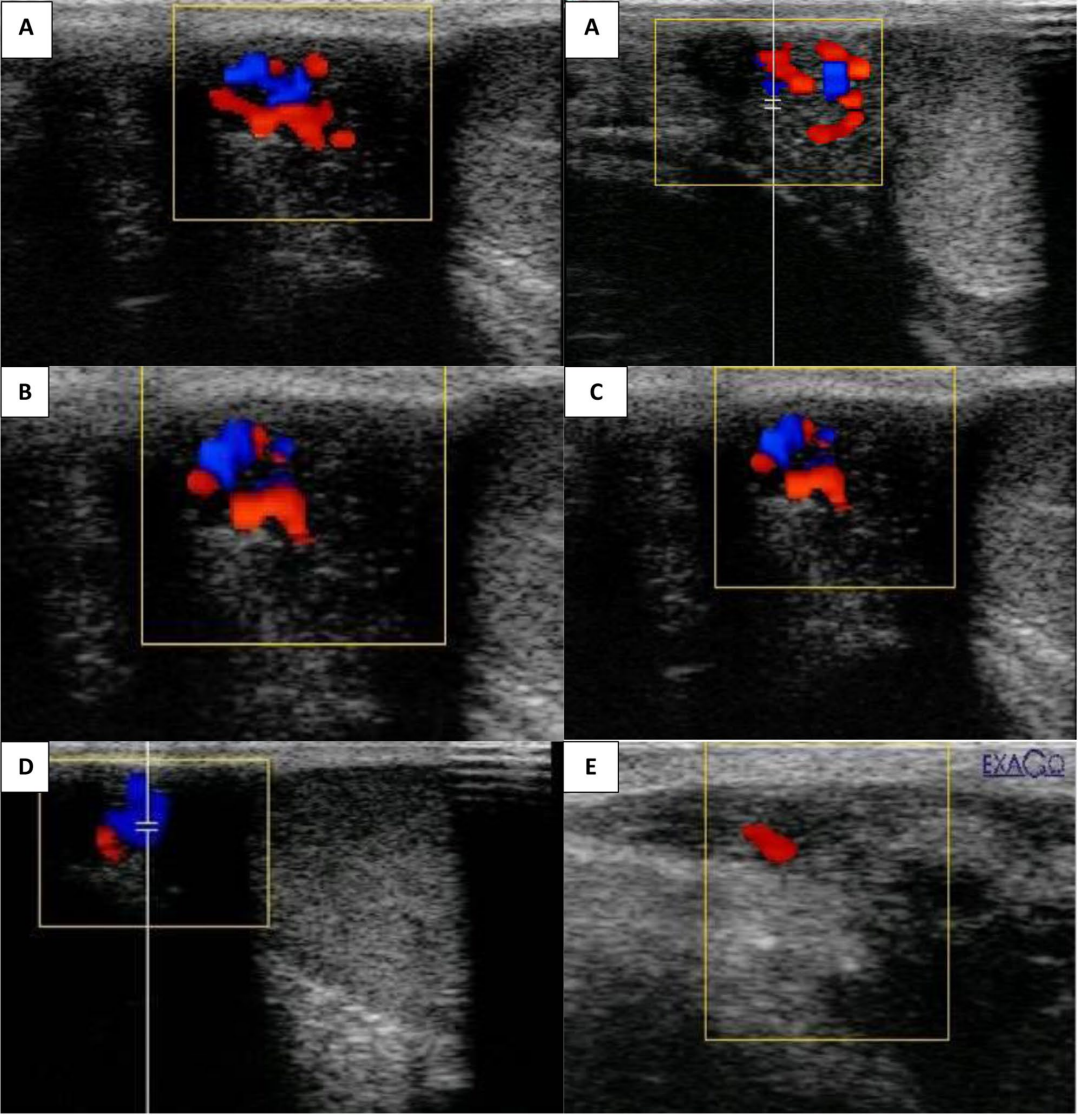
Color Doppler ultrasonography images of the testes before and after injection. The 0, first, second, third and fourth weeks post injection of calcium chloride in dogs with the automatic calculation of both Doppler indices (RI and PI). **A**: Color Doppler ultrasonography of the testes before injection. **B**, **C**, **D** and **E**: First, second, third and fourth weeks after injection, respectively

**Fig. 4:**
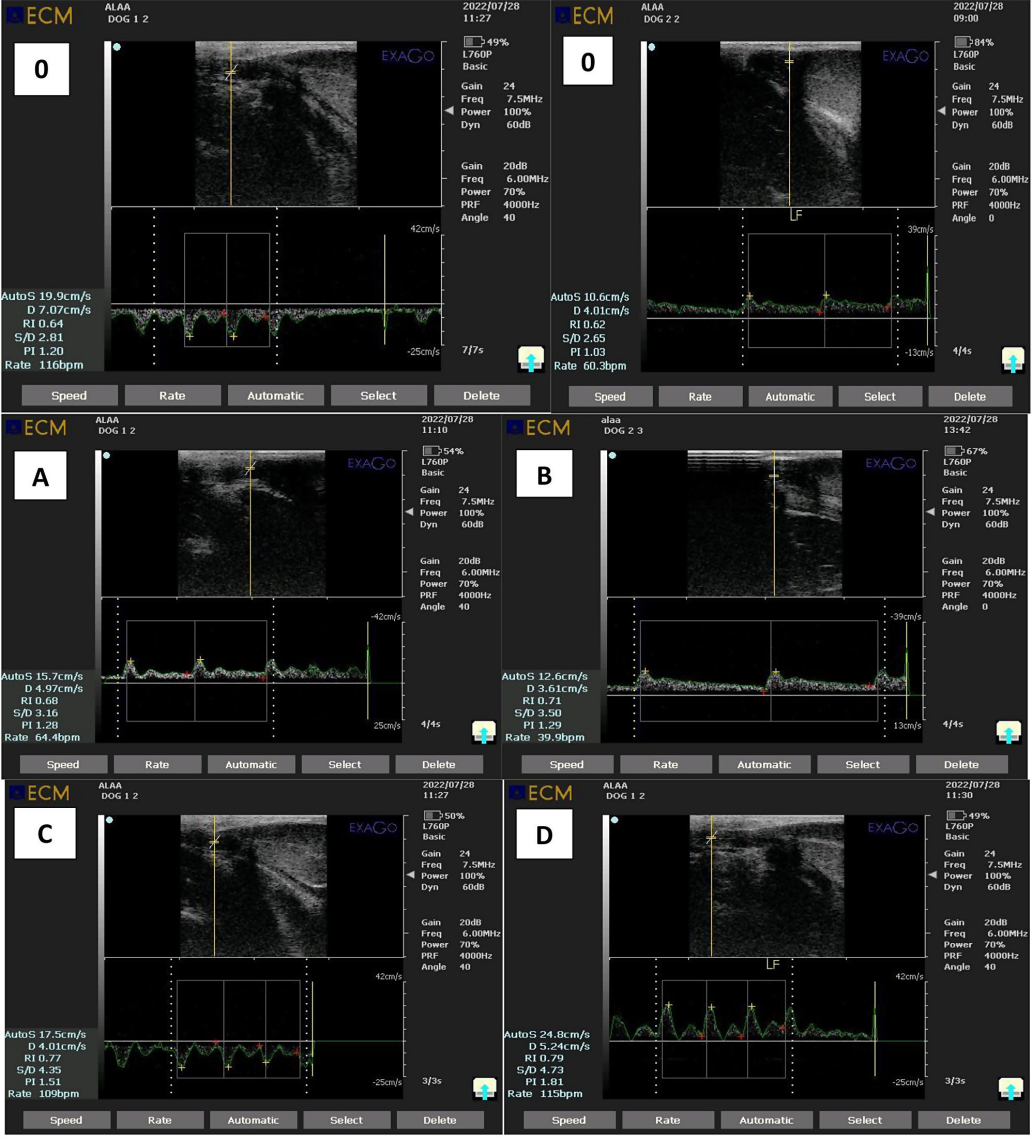
The testicular blood flow with pulsed wave Doppler before and after 0, first, second, third and fourth weeks post injection of calcium chloride in dogs. The yellow star showed the maximum systolic point of velocity (PSV; cm/sec), while the red star showed the end point of velocity due to relaxation (EDV; cm/sec)

A single intratesticular injection of CaCl2 resulted in a significantly higher (*P* < 0.001) resistance index (RI) and pulsatility index (PI) in treated dogs compared to day 0 before CaCl2 injection and controls during weeks 1, 2, 3, and 4. Treated dogs had lower peak systolic velocity (PSV) and end-diastolic velocity (EDV) values (*P* < 0.001) from weeks 1 to 4 (Table 2). Control group PSV and EDV values remained significantly (*P* < 0.01) higher throughout the study period (Figs. 3 and 4).

**Table 2:** Effect of single intratesticular injection of calcium chloride on testicular hemodynamic (Mean ± SEM ) on day 0 before the injection of calcium chloride and 4 weeks post-injection

		Duration of study				
Groups	Measures	Day 0	1st weeks	2nd weeks	3rd weeks	4th weeks
	PSV (cm/sec)	6.23 ± 0.7^ac^	7.45 ± 0.2^ac^	7.45 ± 0.2^ac^	7.65 ± 0.2^ac^	7.45 ± 0.2^ac^
	EDV (cm/sec)	6.24 ± 0.7^ac^	7.45 ± 0.2^ac^	7.45 ± 0.2^ac^	7.64 ± 0.2^ac^	7.45 ± 0.2^ac^
Control	RI	0.62 ± 0.0^ac^	0.63 ± 0.0^ac^	0.63 ± 0.0^ac^	0.62 ± 0.0^ac^	0.63 ± 0.0^ac^
	PI	1.12 ± 0.0^ac^	1.17 ± 0.0^ac^	1.17 ± 0.0^ac^	1.15 ± 0.0^ac^	1.17 ± 0.0^ac^
	PSV (cm/sec)	7.65 ± 0.2^ac^	4.92 ± 0.4^bd^	4.60 ± 0.4^bd^	4.36 ± 0.7^bd^	3.19 ± 0.6^be^
Cacl2	EDV (cm/sec)	7.65 ± 0.2^ac^	4.92 ± 0.4^bd^	4.60 ± 0.4^bd^	4.36 ± 0.7^bd^	3.19 ± 0.6^be^
	RI	0.63 ± 0.0^ac^	0.69 ± 0.0^bd^	0.71 ± 0.0^bd^	0.71 ± 0.0^bd^	0.78 ± 0.0^be^
	PI	1.12 ± 0.0^ac^	1.34 ± 0.0^bd^	1.37 ± 0.0^bd^	1.35 ± 0.0^bd^	1.64 ± 0.0^be^

Mean with different superscripts (a, b) within the same column, (c, d, e) within rows were significantly different at *P* < 0.001

### Semen analysis

CaCl2-treated dogs exhibited a significant (*P* < 0.001) decrease in mean sperm concentration, motility, and live/dead ratio and an increase in abnormal sperm percentage compared to day 0 before CaCl2 injection and control dogs. Azoospermia was observed in treated dogs from week 2 and continued through week 4 (Table 3).

**Table 3:** Effect of single intratesticular injection of calcium chloride on sperm cell concentration (x10^6^/ml), motility%, live /dead (L/D ratio) and Abnormalities % in dogs (Mean ± SEM) on day 0 before the injection of calcium chloride and 4 weeks post-injection

		Duration of study				
Groups	Measures	Day 0	1st weeks	2nd weeks	3rd weeks	4th weeks
	Sperm cell concentration (x10^6^/ml)	219 ± 0.7^a^	219.6 ± 0.5^a^	220.6 ± 0.9^a^	222 ± 0.7 ^a^	221.44 ± 0.5^a^
Control	Motility %	69.6 ± 0.5^a^	70.0 ± 0.7^a^	68.4 ± 1.2 ^a^	70.8 ± 0.7 ^a^	70.6 ± 1.7 ^a^
	L/D ratio	83.6 ± 1.2^a^	86.0 ± 0.7^a^	86.8 ± 0.8 ^a^	84.2 ± 0.8 ^a^	85.0 ± 0.8 ^a^
	Abnormalities%	12.8 ± 0.8^a^	12.0 ± 0.7 ^a^	12.2 ± 0.8 ^a^	12.4 ± 0.9 ^a^	13.0 ± 0.8 ^a^
	Sperm cell concentration (x10^6^/ml)	220.6 ± 0.9^a^	17.7 ± 0.8^b^	azoospermic	azoospermic	azoospermic
Cacl2	Motility %	68.8 ± 0.7^a^	27.4 ± 0.9^b^	-	-	-
	L/D ratio	84.2 ± 1.3^a^	18.0 ± 0.7^b^	-	-	-
	Abnormalities%	12.0 ± 0.7^a^	27.0 ± 0.7^b^	-	-	-

Mean with different superscripts (a, b) within the same column was significantly different at *P* < 0.001

### Serum testosterone levels

Testosterone levels were significantly decreased (*P* < 0.001) in CaCl2-treated dogs compared to day 0 before CaCl2 injection and the control group from weeks 2 to 4 post-injection (Table 4).

**Table 4:** Effect of single intratesticular injection of calcium chloride on the serum level of testosterone in dogs (ng/ml) (mean ± SEM) on day 0 before the injection of calcium chloride and 4 weeks post-injection

		Duration of study				
Groups		Day 0	1st weeks	2nd weeks	3rd weeks	4th weeks
Control		6.3 ± 0.2^ac^	5.9 ± 0.1^ac^	5.9 ± 0.3^ac^	6.0 ± 0.1^ac^	6.0 ± 0.1 ^ac^
Cacl2		6.0 ± 0.1^ac^	5.7 ± 0.2 ^ac^	4.1 ± 0.3 ^bd^	3.7 ± 0.2 ^bd^	2.3 ± 0.2 ^be^

Mean with different superscripts (a, b) within the same column, (c, d, e) within rows were was significantly different at *p* < 0.001

## Discussion

Despite these results, no previous studies have been conducted on the effect of intratesticular CaCl2 injection on testicular hemodynamics. Therefore, the purpose of this study is to look into how male stray dogs’ testicular hemodynamics and semen properties are affected by a single intratesticular injection of CaCl2. In addition to addressing related public health issues, our research may yield important insights for a minimally invasive, effective alternative to surgical sterilization in managing the stray dog population. The idea for the current study came from earlier studies on chemical castration in dogs using intratesticular injections of CaCl2 by Karami et al. [7], which neglected to consider the effect on testicular blood flow. This investigation demonstrates that a single intratesticular injection of CaCl2 effectively reduces the volume, echogenicity, and weight of dog testes. The reason for this decrease appears to be the fibrosis that the CaCl2 caused, which supports results from previous research by Jana and Samanta [8], Karami et al. [7], who reported testicular necrosis after CaCl2 therapy. Because CaCl2 irritates testicular cells, it is thought to trigger an inflammatory response [6].

Testicular necrosis, Leydig cell atrophy, leukocyte infiltration, and fibrosis are among the pathological effects that have been documented in previous investigations [22, 23]. For example, Martins et al. [24] discovered that buffalo bull testes exhibited fibrosis following a 30% CaCl2 injection. Moreover, Canpolat et al. [25] found that pups, adult dogs, and calves treated with 20% and 30% CaCl2 had a notable necrosis of tubules with Leydig cells in the lumen of the seminiferous tubules. The current investigation found that, as compared to vehicle control animals, CaCl2 significantly reduced the concentration, motility, and viability of sperm in dog semen. After two weeks of injection, azoospermia was seen, and it lasted for the end of the study. This condition was probably caused by coagulative necrosis of the seminiferous tubules. These findings are consistent with those of Karami et al. [7], which reported a decrease in sperm count in the canine testes after CaCl2 injection [8]. CaCl2 degrades spermatogonial stem cells over time, resulting in necrotic germ cells and the incapacity of the seminiferous tubules to commence spermatogenesis again [26].

B-mode Ultrasonography evaluation of testes revealed that dogs in the control group had homogenous, medium echotextures and seemed echogenic in their testes. A thin, hyperechoic peripheral echo was formed by the parietal and visceral tunics. On the midsagittal plane, the mediastinum testis is evident as an echogenic central linear structure, and on the mid-transverse scan plane, it is apparent as a central focal echo. This finding was described by Zappone et al. [27]. On the other hand, testicular sonography in the treated group revealed distinct localized hyperechoic regions in the parenchyma. In addition, there were anechoic areas all around the hyperechoic ones. According to a recent study by Leoci et al. [28], these anechoic locations corresponded to tissue damage areas caused by the CaCl2 injection. The sclerosed parenchyma tissues likely caused these hyperechoic areas to become increasingly echogenic.

The current investigation also found significant inflammation and fibrosis in the interstitial spaces, which is in line with earlier findings [29]. In comparison to controls, the current study’s findings on testosterone levels showed a substantial drop after therapy. Previous studies Jana and Samanta, Abu-Ahmed [8, 30] support the attribution of this drop to the degradation of Leydig cells caused by CaCl2. According to Chainy et al. [31], the production of free radicals in the testicular tissue is probably the cause of the drop in testosterone levels. Some investigations, however, have found no discernible change in testosterone levels following injection, which runs counter to these findings [7, 12].

To our knowledge, this is the first study evaluating the impact of a single intratesticular injection of CaCl2 on testicular hemodynamics in dogs. The findings corroborate the theory that the injection in question reduces the perfusion of the testicular vessels, as demonstrated by decreased end-diastolic and peak systolic velocities (PSV) and elevated resistive and pulsatility indices (RI and PI). These results imply that CaCl2 is a good non-invasive substitute for surgical castration in canines of the male gender. A lower testicular blood flow was linked to an increase in leukocytes in blood vessels, which in turn caused inflammatory responses in these locations. This might be the cause of the drop in EDV and PSV that was accompanied by an increase in RI and PI. Leukocytes may cause additional oxygen radical-mediated damage to the tissue [32]. Since the testicles have a special vascular structure with low intra-testicular capillary pressure and high flow resistance, any change in blood flow can cause significant functional changes. This makes controlling testicular blood flow extremely important [10, 33, 34]. Further studies are needed to explore the effect of a single intratesticular of CaCl2 on the oxidative stress and antioxidant capacity of the testes, which affect directly the process of spermatogenesis.

## Conclusion

This study provides evidence that a single intratesticular injection of CaCl2 in dogs significantly impacts testicular volume, pathology, blood flow and function, with implications for its use as a chemical castration method.

## Data Availability

Data is provided within the manuscript.
